# The Case for a Hippocratic Oath for Connected Medical Devices: Viewpoint

**DOI:** 10.2196/12568

**Published:** 2019-03-19

**Authors:** Beau Woods, Andrea Coravos, Joshua David Corman

**Affiliations:** 1 I Am The Cavalry Washington, DC United States; 2 Biohacking Village New York, NY United States; 3 Atlantic Council Washington, DC United States; 4 Elektra Labs Boston, MA United States; 5 Harvard-MIT Center for Regulatory Science Harvard University Boston, MA United States; 6 Digital Medicine (DiMe) Society New York, NY United States; 7 Heinz College of Information Systems and Public Policy Carnegie Mellon University Pittsburgh, PA United States; 8 PTC Needham, MA United States

**Keywords:** ethics, cybersecurity, information technology, delivery of health care, connected devices

## Abstract

Prior to graduating from medical school, soon-to-be physicians take the Hippocratic Oath, a symbolic declaration to provide care in the best interest of patients. As the medical community increasingly deploys connected devices to deliver patient care, a critical question emerges: should the manufacturers and adopters of these connected technologies be governed by the symbolic spirit of the Hippocratic Oath? In 2016, I Am The Cavalry, a grassroots initiative from the cybersecurity research community, published the first Hippocratic Oath for Connected Medical Devices (HOCMD), containing 5 principles. Over the past three years, the HOCMD has gained broad support and influenced regulatory policy. We introduce 5 case studies of the HOCMD in practice, illustrating how the 5 principles can lead to a safer and more effective adoption of connected medical technologies.

## Introduction

Prior to graduating from medical school, soon-to-be physicians take the Hippocratic Oath [1], a symbolic declaration to provide care in the best interest of patients. As the medical community increasingly deploys connected devices to deliver patient care, a critical question emerges: should the manufacturers and adopters of these connected technologies be governed by the symbolic spirit of the Hippocratic Oath?

**Figure 1:**
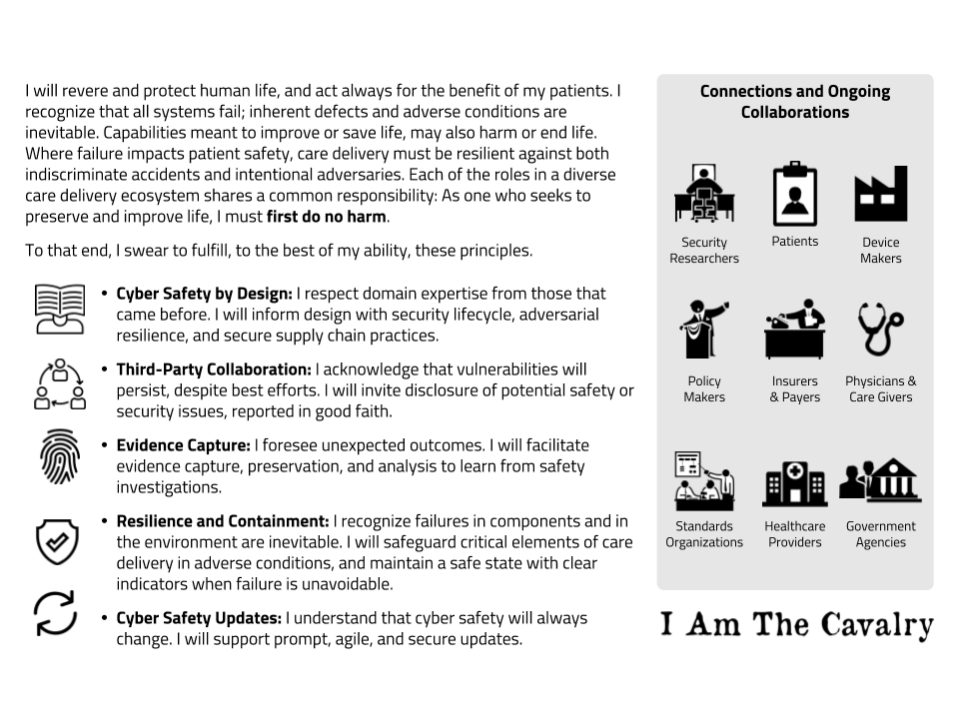
Hippocratic Oath for Connected Medical Devices (HOCMD).

Five years ago, Joshua David Corman and Nicholas Percoco founded *I Am The Cavalry*, a grassroots initiative from the cybersecurity research community. Its mission is “to ensure technologies with the potential to impact public safety and human life are worthy of our trust” [2]. In 2016, along with a diverse stakeholder group across the health care ecosystem, including payers, providers, patients, policy makers, and physicians, I Am The Cavalry drafted the first version of a Hippocratic Oath for Connected Medical Devices (HOCMD) (see Figure 1) and published an open letter on its website [3]. Similar to graduating medical students who pledge to protect the patient and hospital systems who pledge to protect patients’ data and information, the HOCMD outlines five guiding ethical principles for manufacturers, organizations, and individuals delivering care through connected medical devices.

Adopting these principles not only builds trust in the connected product for patients, clinicians, hospitals, and regulators, but also serves a business function. Similar to a drug that is pulled off the market because of unintended side effects, connected products with security vulnerabilities can sharply impact a company’s share price and valuation. For medical device investors who want to avoid security issues in their portfolio companies, these principles enable them to evaluate a connected medical product’s risk profile, safety, and effectiveness.

Although all systems eventually fail, not all failures have to cause harm. Organizations that follow these five principles tend to build safer products with fewer malfunctions and deliver better care to patients. To illustrate the principles, we have outlined five case studies based on true events; we have blinded some examples at the request of the parties involved.

## Five Case Studies based on the 5 Oath Principles

### Oath Principle #1: Cyber Safety by Design

Riley, a hospital procurement analyst, reviews several medical devices, struggling to distinguish security features between similar products and the potential impact on cost and risk to her organization. Riley and her peers often rely on sales literature and expensive internal testing to understand how to buy the best devices to fit their clinical environments. Products with better documentation make her job easier and increase the organization’s confidence that the devices can be deployed and used safely and effectively, without undue cost or risk.

Riley prefers to procure devices from manufacturers who follow practices laid out in *cyber safety by design*, because she can more easily anticipate and avoid product failures. For example, preferred devices would have instructions for safe and secure implementation, design assumptions, a *software bill of materials* (SBOM), and other documentation outlining how cybersecurity is designed into the product’s lifecycle. Health care organizations like the Mayo Clinic have started requiring manufacturers to disclose more information about their security capabilities [4]. These requirements are being widely adopted by manufacturers like Philips, Siemens, and Becton Dickinson, and are becoming standard practice through industry-led initiatives like the Manufacturer Disclosure Statement for Medical Device Security (MDS2) disclosures [5].

This past year, the US Food and Drug Administration (FDA) incorporated the *cyber safety by design* principles in their Medical Device Safety Action Plan [6] and updated Premarket Guidance [7], which proposes requiring an SBOM or *cybersecurity bill of materials* (CBOM), in which manufacturers have to clearly communicate choices and capabilities that impact cyber safety and other practices from this first principle.

### Oath Principle #2: Third-Party Collaboration

In early 2017, Jay Radcliffe, a researcher with the security firm Rapid7, discovered a flaw in how the Johnson & Johnson Animas insulin pumps handle commands from the unit’s remote control. Exploiting the flaw could potentially lead to unauthorized access to the pump through its unencrypted radio frequency communication system.

Undisclosed flaws represent potential harm to patients through accidents or adverse events. While it may seem inconceivable, many manufacturers have threatened security researchers with legal repercussions [8] for identifying these kinds of issues. Although immediate public disclosure often catalyzes a prompt fix, releasing the disclosure often starts a footrace between defenders and adversaries that may put patients at greater risk. Jay had a difficult decision to make: should he disclose the vulnerability and, if so, how?

Thankfully, Johnson & Johnson had recently drafted a coordinated disclosure policy that invited third-party collaboration. After investigation, the issue was considered to be a low safety risk due to existing mitigations. Jay and Johnson & Johnson codeveloped an effective approach to addressing the issue, communicated with patients and physicians, kept the FDA and other organizations informed, and improved internal processes. This was a win for all involved and decreased potential harm to patients.

Many throughout the health care ecosystem, including regulators like the FDA, are voicing their support for coordinated vulnerability disclosure. The FDA recently outlined this principle in their guidance for Post-Market Management of Cybersecurity in Medical Devices [9] and published a collaborative report with the Medical Device Innovation Consortium (MDIC) that advances the concept of coordinated disclosure [10]. Perhaps untraditionally, the FDA has also started to participate at hacker conferences like DEF CON [11] to encourage greater collaboration when surfacing vulnerabilities.

As of this writing, nearly 20 companies have published programs [12] to receive and handle reports of security vulnerabilities. Programs based around standards (ie, ISO 29147, ISO 30111, and National Telecommunications and Information Administration [NTIA] Early Stage Template [13]) frequently leverage existing mechanisms internally that are well-tested and focus on incentives, aligning researchers and device makers toward safer products.

### Oath Principle #3: Evidence Capture

Two years ago, a hospital sent a life-critical device to the manufacturer for investigation, suspecting that it was tampered with by a physician who was being sued for malpractice. After conducting a review, the manufacturer discovered that evidence of patient care had been wiped from the device, but it was unclear who did this, when, or why. The connected device lacked forensically sound evidence-capture tools.

When doctors are not sure why an adverse event happened, they perform tests or, in the case of death, an autopsy. When medical devices are involved, evidence from the device can and should support postmortem investigations as part of that autopsy. For this reason, the third principle of the Oath, *evidence capture*, alerts manufacturers to the need for built-in evidence-capture capabilities and tamper-evident logging to protect patients.

For instance, a secure system could store a fixed record of safety- and security-related data, such as software integrity checks and activity logs. This information could then be reviewed by analysts at the health care organization or the manufacturer to support investigations into malicious tampering, accidental harm, or cybersecurity issues. In addition, this capability lays the foundation for future capabilities that allow for more prompt and agile responses.

The FDA’s recent draft premarket guidance [7] calls for a forensically sound evidence-capture capability, despite relatively little interest from industry and regulatory bodies. This capability would be invaluable; most devices currently lack activity logs and other integrity checks, indicating that it is possible that there have been adverse events related to device malfunction of which manufacturers are unaware. The FDA’s updated guidance will likely incentivize more device makers to include this capability in their products.

### Oath Principle #4: Resilience and Containment

In 2017, several German journalists sat in a room learning how to hack medical devices at a press event held by Dräger. First, they learned how to use tools to penetrate a basic installation of Windows XP, an operating system that had been unsupported for three years. After gaining complete control of the ventilators, the journalists tried similar techniques against a version of Windows XP that Dräger uses in some of its current products. None of the journalists, nor the subsequent professional penetration testers, were able to hack the device over the network. The company had hardened the system by disabling nonessential functions, encrypting data in transit, and isolating the network-connected elements from those that deliver patient care.

Medical devices are increasingly connected to the rest of the hospital and to the Internet. A traditional stethoscope or ultrasound are not connected to each other, let alone exposed to technically savvy adversaries thousands of miles away. Practices in the *resilience and containment* principle can contain the reach of adversaries by reducing risk from cascading cybersecurity failures, making failures evident, and ensuring devices default to a safe mode when they must fail.

Building on the *resilience and containment* principle, the FDA’s premarket guidance outlines best practices, such as improving device isolation using firewalls, reducing elective exposure by disabling network-connected components, and protecting patient record integrity by encrypting data while in transit and on the device. The FDA has also issued two safety notifications related to failure to segregate safety-critical from noncritical components of Hospira infusion pumps [14,15].

### Oath Principle #5: Cyber Safety Updates

In 2016, a vulnerability was discovered in St. Jude Medical’s pacemakers, implantable cardioverter defibrillators, and bedside monitors. The chances that an adversary would exploit this vulnerability to cause harm seemed low, but the attack could be carried out stealthily, triggering heart conditions in patients. An estimated 465,000 patients would be at risk before the manufacturer could build replacements and doctors could implant them [16].

Fortunately, St. Jude Medical had a better way to address the issue. A series of three software updates greatly reduced cost, increased adoption, hastened correction timelines, and minimized side effects compared to extreme solutions, such as replacing the device through explantation. A study showed that 25% of patients had the software update applied at their next clinic visit, with no pacing failures observed [17].

It should be common practice to update software routinely. The FDA has repeatedly stated that manufacturers are responsible for delivering security updates, which are considered routine and therefore not often subject to additional reviews. However, security and promptness of these updates still lag for most device makers and communication is difficult with many stakeholders involved with each device.

## Accelerating Adoption of the Hippocratic Oath for Connected Medical Devices

While most doctors practicing in the United States take the Hippocratic Oath before going into clinical practice, developers of software-connected devices traditionally do not share a similar gatekeeper. As of today, individuals and organizations can publicly commit to the HOCMD via the I Am The Cavalry website [3]. Though this lacks the resonance of repeating the Oath aloud at a hard-earned graduation, the digital pledge carries its own gravitas. Digital statements are accessible and searchable. An online pledge holds leadership, scientists, and engineers accountable for their work by the public and signals an unwavering faith in the quality of the products they have created. The Oath can also be taken by organizations, like hospital systems, who pledge to act not only in the best interest of the corporal patient, but also in the best interest of the patient’s information—the patient’s *digital specimen*.

Oaths can serve two important purposes: first, to establish a set of standards and second, to remind an oath-taking community of their promised commitments. Although oaths have a storied history, evidence does not necessarily suggest that taking an oath leads to better behavior in practice even within medical professions [18]. Thus, as the HOCMD matures, its principles may be deployed more effectively in other formats. For instance, a checklist may be a more effective format than an oath for putting the principles into routine practice [19]. Atul Gawande popularized practical checklists in health care when he published The Checklist Manifesto, a short book on how not to make big mistakes, and outlined how a checklist format can increase efficiency, consistency, and safety [20].

The HOCMD’s influence on regulatory policy is another example of its principles moving from theory to practice. Although the FDA draft guidance for Management of Cybersecurity in Medical Devices, published in October 2018, does not call out the Oath by name, it references four Oath principles in its premarket guidance [7] and references the fifth in its postmarket guidance [9]. FDA officials have shared in speeches [11] and tweets [21] their learnings from the security and hacker communities as they draft modern policies to bring safe, connected, medical products to market.

We envision a world where all medical device manufacturers stand proudly behind their work by publicly committing to upholding these principles of cyber safety; we also envision a world where patients, hospitals, doctors, insurers, and regulators strongly favor more secure medical devices built by manufacturers who align with the Oath. As software-driven connected products drive more care delivery, we hope that those who manufacture and adopt life-critical products will commit to carrying similar values as the physicians who have attended to patients for centuries.

## Abbreviations

CBOM: cybersecurity bill of materials
FDA: US Food and Drug Administration
HOCMD: Hippocratic Oath for Connected Medical Devices
MDIC: Medical Device Innovation Consortium
MDS2: Manufacturer Disclosure Statement for Medical Device Security
NTIA: National Telecommunications and Information Administration
SBOM: software bill of materials
